# Proton-activated chloride channel PACC1 as acid sensor in epidermal desquamation

**DOI:** 10.1073/pnas.2601316123

**Published:** 2026-03-31

**Authors:** Keyu Xia, Xiangjian Liu, Jiajing Wu, Junyan Hu, Xufeng Cheng, Tingyan Mi, Bomin Gao, Xiaoyi Mo, Xuteng Lu, Feng Cao, Chang Xie, Jian Cao, Nadine Pernodet, Guangwen He, Huanjun Zhou, Jing Yao

**Affiliations:** ^a^State Key Laboratory of Virology and Biosafety, Hubei Provincial Research Center for Basic Biological Sciences, College of Life Sciences, TaiKang Center for Life and Medical Sciences, Hubei Key Laboratory of Cell Homeostasis, Frontier Science Center for Immunology and Metabolism, Wuhan University, Wuhan 430072, Hubei, China; ^b^Estée Lauder Companies Innovation R&D (China) Co. Ltd., Shanghai 200233, China; ^c^Global Research and Development, The Estée Lauder Companies, Melville, NY 11747

**Keywords:** PACC1, epidermal exfoliation, acid sensation, skin renewal

## Abstract

The acidic microenvironment of the stratum corneum is crucial for epidermal desquamation and barrier homeostasis, yet the primary proton sensor that triggers this process remains unknown. Here, we report that the proton-activated chloride channel PACC1 is essential for acid-induced upregulation of kallikreins (KLKs) and desmosomal degradation, two key steps in skin exfoliation. Functional and protein expression analyses revealed that PACC1 is the predominant acid-sensitive ion channel in keratinocytes. Proton-mediated PACC1 activation evokes chloride efflux and initiates a signaling cascade via the c-Jun N-terminal Kinase/AP-1 (JNK/AP-1) pathway. This cascade significantly enhances the expression and secretion of KLKs (KLK5/7), thereby facilitating desquamation through corneodesmosomal degradation. Notably, acid-induced KLK upregulation was abolished by PACC1 knockdown, knockout, or mutants that are deficient in proton sensing. This effect was also observed with pharmacological channel inhibition and was specifically restored by reconstitution with functional PACC1. These findings establish PACC1 as the core sensor that converts epidermal acidification into a desquamation signal, providing a mechanistic foundation for developing targeted therapeutic and cosmetic strategies that modulate skin barrier function.

The skin consists of epidermis, dermis, and subcutaneous tissue. It serves as a vital barrier against environmental insults while maintaining hydration and integrity (1). The stratum corneum, the outermost hydrophobic layer of the epidermis, forms the primary defensive barrier. This barrier is maintained by continuous keratinocyte turnover, which involves proliferation in the basal layer, differentiation and migration upward, and regulated exfoliation of corneocytes (2). Recent studies have delineated a sophisticated stepwise pH zonation within the stratum corneum, critical for barrier homeostasis (3, 4).

Exfoliation is tightly controlled by a pH-sensitive proteolytic system. Kallikreins (KLKs) degrade desmosomal proteins and release corneocytes. Conversely, the lymphoepithelial Kazal-type-related inhibitor (LEKTI) suppresses KLK activity in the deeper stratum corneum. As the pH level decreases toward the skin surface, LEKTI–KLK complexes dissociate, thereby activating desquamation (5). Dysregulated KLK activity, which is often associated with altered skin pH, is a key factor in the development of diseases such as atopic dermatitis and psoriasis (6). Chemical exfoliation via acids is also a common clinical intervention (7). Although acidic pH was previously shown to activate chloride channels in keratinocytes (8), the molecular identity of the primary sensor has remained elusive.

Here, we identified PACC1 as the primary acid sensor in human keratinocytes. Knockout and pharmacological inhibition of PACC1 confirmed that PACC1 is necessary for the acid-induced KLK5/7 upregulation and secretion, processes that rely on PACC1' s proton-sensing capability and ion-conducting function. Our data further revealed that PACC1 mediates acid-sensing signals through the JNK/AP-1 pathway to regulate KLK transcription. Together, our findings delineate the key signaling axis that drives acid-induced exfoliation, establishing PACC1 as a core regulator of epidermal homeostasis and a promising therapeutic target.

## Results

**Fig. 1.**
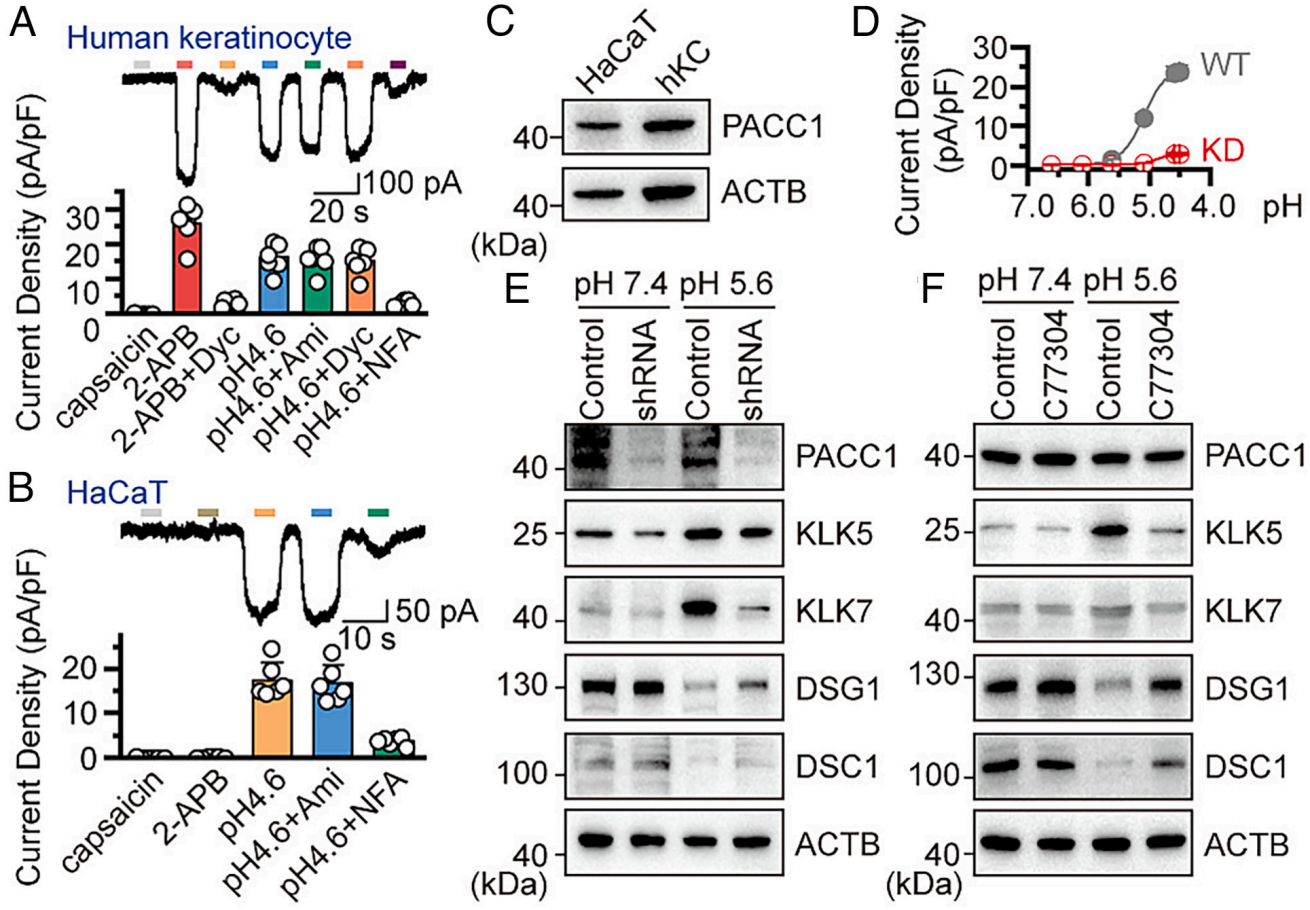
PACC1 functions as an essential proton-sensing channel in regulating acid-induced skin desquamation. (*A*) Representative whole-cell recordings in human keratinocytes. Dyc: Dyclonine; Ami: Amiloride; NFA: Niflumic Acid. (*B*) Parallel recordings in HaCaT cells. (*C*) Expression level of PACC1 in HaCaTs and Human keratinocytes. (*D*) Representative whole-cell currents evoked by varying pH gradients in PACC1-KD (red) and WT (gray) HaCaT cells. (*E*) Expression levels of PACC1, KLK5, KLK7, DSG1, and DSC1 in WT and PACC1-KD HaCaT cells under differential pH conditions. (*F*) Expressions of PACC1, KLK5, KLK7, DSG1, and DSC1 in HaCaT cells under differential pH conditions with or without 300 μM C77304. Data are representative of three independent experiments (*C*, *E*, and *F*), and the corresponding original images with densitometric analyses are available in Dataset S2.

The epidermal pH gradient promotes desquamation by dissociating LEKTI–KLK complexes in the stratum corneum, a process fundamental to barrier homeostasis and leveraged in skin care (5–7). However, the molecular sensor that enables keratinocytes to detect extracellular acidification remains undefined. To identify this sensor, we performed patch-clamp recordings in human primary keratinocytes and HaCaT cells. As illustrated in Fig. 1 *A* and *B*, acid (pH 4.6) alone produced a strong response. However, coapplication with the chloride channel blocker niflumic acid (100 μM) significantly reduced the acid-induced currents in both human keratinocytes and HaCaTs (Fig. 1 *A* and *B*). This proton-activated current was unaffected by agonists or antagonists of several known acid-sensitive ion channels (e.g., ASIC, TRPV1, and TRPV3). PACC1 has been identified as a proton-activated chloride channel, playing a broad role in the sensation of acidic stimuli and subsequent signal transduction (9). As shown in Fig. 1*C*, Western blot analysis revealed the abundant expression of PACC1 in both cell types.

To investigate the functional role of PACC1 in the exfoliation pathway, we examined whether its activation links acid sensing to the expression of important exfoliation enzymes, especially KLKs. Previous studies have demonstrated that acidic exfoliation involves KLK proteases, desmosomal components, and LEKTI (6). We generated a PACC1 knockdown cell line and confirmed the knockdown efficacy by electrophysiological analysis (Fig. 1*D*). After 2 h of stimulation at pH 5.6 and 24 h of culture, the levels of KLK5/7 proteins increased, while the levels of DSG1 and DSC1 desmosomal proteins decreased in control cells. These acid-induced changes were largely reversed in PACC1 knockdown cells (Fig. 1*E*), confirming that PACC1 specifically couples extracellular acidification to the regulation of exfoliation-related proteins.

To determine whether PACC1 channel activity is necessary for downstream signaling, we used C77304 (10), a selective PACC1 antagonist, to pharmacologically inhibit it. Western blot analysis revealed that the increase in KLK5/7 protein levels induced by acid (pH 5.6) in HaCaT cells was significantly reduced by cotreatment with 300 μM C77304 (Fig. 1*F*). The inhibitor also reversed the reduction in desmosomal components DSG1 and DSC1 mediated by acid. These results suggest that PACC1-mediated chloride conductance, rather than the mere presence of the protein, is crucial for converting the acidic signal into KLK gene expression and subsequent desmosomal degradation.

**Fig. 2.**
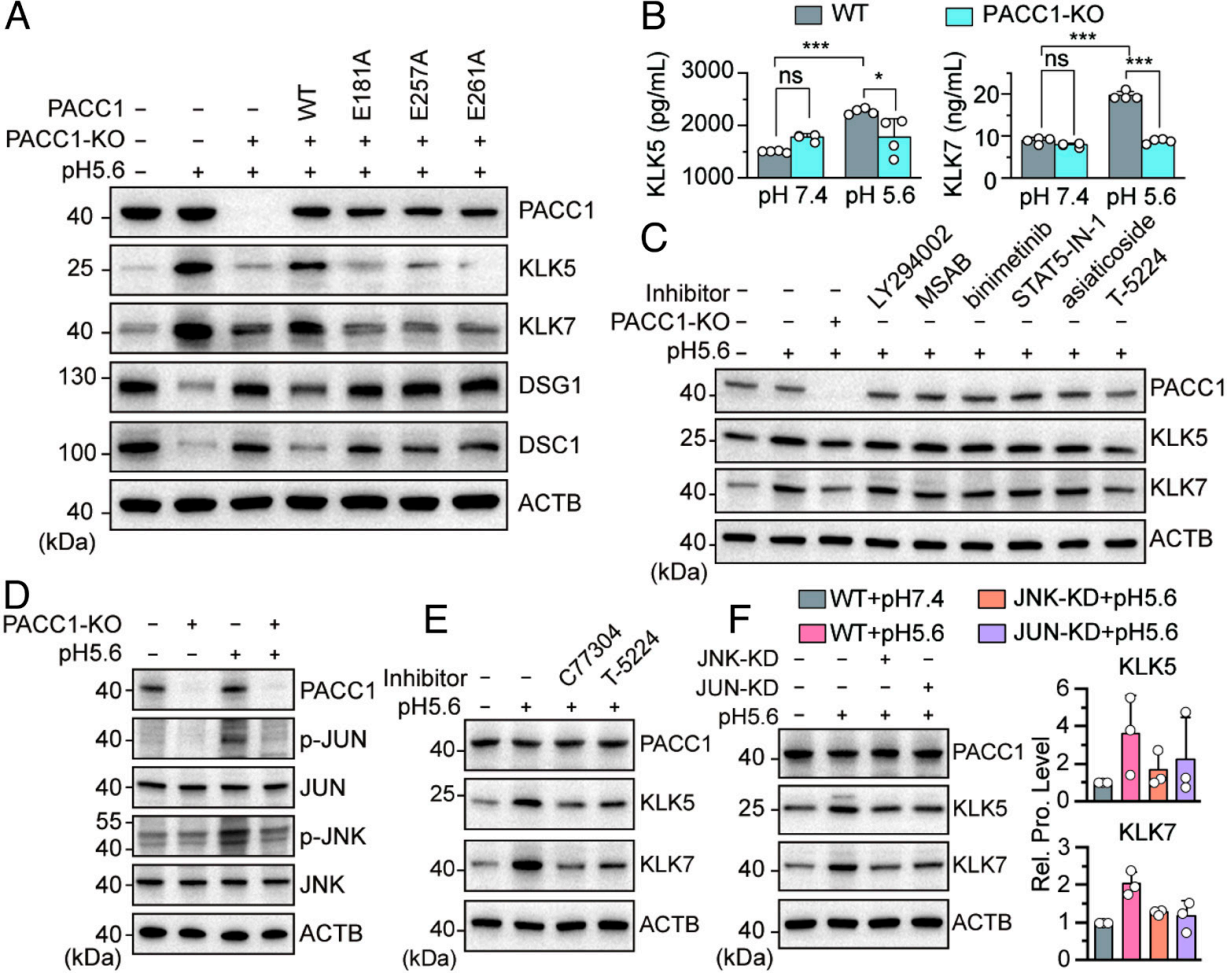
PACC1 mediates acid-induced KLK expression and desmosomal protein cleavage via its proton-sensing channel activity and JNK/AP-1 signaling. (*A*) Expressions of PACC1, KLK5, KLK7, DSG1, and DSC1 in WT and PACC1 knockout cells under acidic stress. Three mutants (E181A, E257A, and E261A) were included for functional validation. (*B*) ELISA analysis of KLK5/7 in WT and PACC1-KO HaCaT cells at differential pH conditions. (*C*) Impact of various signaling pathway inhibitors on the acid effect in HaCaT cells. (*D*) Effects of acidic stimulation and PACC1 on the phosphorylation of JUN and JNK. (*E*) Effects of C77304 and T-5224 on the protein levels of KLK5/7 in human keratinocytes. (*F*) Expressions of PACC1 and KLK5/7 in JUN- and JNK-knockdown human keratinocytes under differential pH conditions. Data are representative of three independent experiments (*A* and *C*–*F*), and the corresponding original images with densitometric analyses are available in Dataset S2.

To confirm the essential role of PACC1’s proton-sensing ability in exfoliation, we created PACC1-knockout HaCaT cells using the CRISPR/Cas9 technique. Reconstitution with WT PACC1 restored acid-induced KLK expression. However, proton-sensing mutants (E181A, E257A, and E261A) did not, suggesting that the ability to detect extracellular protons is necessary for PACC1-mediated KLK upregulation (Fig. 2*A*) (10). Consistent with KLKs functioning as secreted proteases, Enzyme-Linked Immunosorbent Assay (ELISA) measurements showed that acid stimulation increased the level of extracellular KLKs in control cells. This effect was markedly reduced in PACC1-knockout cells (Fig. 2*B*). Together, these data demonstrate that PACC1 activation via its proton-sensing residues is necessary for KLKs upregulation and secretion in response to extracellular acidification.

To identify the signaling pathways that mediate acid-induced KLK upregulation, we screened pathway inhibitors. Only T-5224, an AP-1 inhibitor, substantially blocked the acid-induced increase in KLK5/7 protein levels, in contrast to the inhibitors targeting PI3K-Akt (LY294002), IL-17 (methyl 3-(4-methylphenylsulfonamido)benzoate, MSAB), MEK/ERK (Binimetinib), Signal Transducer and Activator of Transcription 5 (STAT5-IN-1), and TGF-β/Smad (Asiaticoside) (Fig. 2*C*). This finding implicates AP-1 as a key mediator. Consistent with this finding, acidic stimulation markedly increased JNK and c-Jun (JUN) phosphorylation in wild-type (WT) HaCaT cells. This effect was largely abolished in PACC1-knockout cells (Fig. 2*D*), confirming that acid-induced JNK/AP-1 activation depends on PACC1. Pharmacological inhibition of PACC1 (C77304) or AP-1 (T-5224) during acid stimulation blocked KLK5/7 upregulation (Fig. 2*E*). Furthermore, JNK or JUN knockdown abolished acid-induced KLK induction (Fig. 2*F*), establishing both kinases as essential downstream effectors of PACC1 in this pathway.

Taken together, our findings suggest that PACC1 activates the JNK/AP-1 signaling pathway, thereby mediating acid-induced KLK upregulation in keratinocytes.

## Discussion

This study identifies PACC1, a proton-activated chloride channel, as the key proton sensor in human keratinocytes that translates extracellular acidification into a prodesquamatory signal. We delineate a signaling pathway in which acid activates PACC1, resulting in chloride efflux that initiates a JNK/AP-1-dependent transcriptional program. This cascade ultimately upregulates and promotes the secretion of kallikrein-related peptidases (KLK5/7), leading to keratinocyte exfoliation.

This study proposes a paradigm shift in skin desquamation management. Unlike conventional chemical exfoliants, which broadly lower the pH level and risk disrupting the skin barrier, precise PACC1 activation can directly engage the physiological acid sensor to regulate corneocyte shedding. This minimizes off-target effects and collateral damage to keratinocytes. This strategy provides a safer and more controlled approach for dermatological applications. Furthermore, PACC1 exhibits a unique signaling mechanism. Its activation initiates a direct downstream cascade that couples environmental acid sensing to transcriptional regulation. This expands the framework of cellular signal transduction by establishing channel-mediated sensing as a direct route to controlling gene expression.

The prevailing model for pH-regulated desquamation centers on the direct control of KLK proteases via LEKTI dissociation in the acidic stratum corneum. We propose an additional layer operative in living keratinocytes, where the PACC1–JNK–AP-1 axis senses declining extracellular pH and upregulates KLK5/7 expression. Then KLK5/7 degrade corneodesmosomal proteins, such as DSG1 and DSC1 (11). This PACC1-mediated transcriptional priming represents a complementary pathway to the established LEKTI–KLK regulatory process.

While our 2D culture systems do not fully recapitulate epidermal cornification, consistent findings in HaCaT and primary keratinocytes validate this fundamental mechanism. In the future, it will be interesting to use organotypic 3D cultures and human skin samples to corroborate the PACC1 expression and function in the stratified epidermis. In addition, the precise mechanism linking PACC1 chloride efflux to JNK phosphorylation remains to be elucidated, possibly involving a direct relief of Cl^−^-dependent inhibition on upstream JNK kinases (12), mechanosensitive signaling cascades (13), and divalent cation (e.g., Ca^2+^, Mg^2+^) homeostasis (14–15).

In summary, these findings establish the PACC1 channel as a key player in the acid-induced prodesquamation pathway, advancing the theory of skin barriers and identifying it as a potential therapeutic target for regulating desquamation.

## Materials and Methods

This study used human primary keratinocytes and HaCaT cells. Details for the methods are provided in *SI Appendix*.

## Supplementary Material

Appendix 01 (PDF)

Dataset S01 (XLSX)

## Data Availability

All study data are included in the article and/or supporting information.

## References

[1] V. Haydont, B. A. Bernard, N. O. Fortunel, Age-related evolutions of the dermis: Clinical signs, fibroblast and extracellular matrix dynamics. Mech. Ageing Dev. **177**, 150–156 (2018). 29548941 10.1016/j.mad.2018.03.006

[2] A. V. Rawlings, C. R. Harding, Moisturization and skin barrier function. Dermatol. Ther. **17**, 43–48 (2004). 14728698 10.1111/j.1396-0296.2004.04s1005.x

[3] K. Fukuda , Three stepwise pH progressions in stratum corneum for homeostatic maintenance of the skin. Nat. Commun. **15**, 14062 (2024). 10.1038/s41467-024-48226-z PMC11096370 38750035

[4] K. Fukuda, Y. Ito, M. Amagai, The acid mantle reimagined: Unveiling the role of stepwise pH zonation in the stratum corneum. J. Invest. Dermatol. **145**, 2147–2152 (2025). 40057859 10.1016/j.jid.2025.02.129

[5] E. Delva, D. K. Tucker, A. P. Kowalczyk, The desmosome. Cold Spring Harb. Perspect. Biol. **1**, a002543 (2009). 20066089 10.1101/cshperspect.a002543 PMC2742091

[6] J. Chavarria-Smith , Dual antibody inhibition of KLK5 and KLK7 for Netherton syndrome and atopic dermatitis. Sci. Transl. Med. **14**, eabp9159 (2022). 36516271 10.1126/scitranslmed.abp9159

[7] Ș. E. MăgeruȘan, G. Hancu, A. Rusu, A comprehensive bibliographic review concerning the efficacy of organic acids for chemical peels treating Acne Vulgaris. Molecules **28**, 7219 (2023). 37894698 10.3390/molecules28207219 PMC10608815

[8] S. J. Park , Acidic pH-activated Cl^−^ current and intracellular Ca^2+^ response in human keratinocytes. Korean J. Physiol. Pharmacol. **12**, 177–183 (2008). 19967053 10.4196/kjpp.2008.12.4.177 PMC2788633

[9] J. Yang , PAC, an evolutionarily conserved membrane protein, is a proton-activated chloride channel. Science **364**, 395–399 (2019). 31023925 10.1126/science.aav9739 PMC7305803

[10] P. Zhao , A new polymodal gating model of the proton-activated chloride channel. PLoS Biol. **21**, e3002309 (2023). 37713449 10.1371/journal.pbio.3002309 PMC10529583

[11] J.-I. Sakabe , Kallikrein-related peptidase 5 functions in proteolytic processing of profilaggrin in cultured human keratinocytes. J. Biol. Chem. **288**, 17179–17189 (2013). 23629652 10.1074/jbc.M113.476820 PMC3682523

[12] J. M. Capasso, C. J. Rivard, L. M. Enomoto, T. Berl, Chloride, not sodium, stimulates expression of the γ subunit of Na/K-ATPase and activates JNK in response to hypertonicity in mouse IMCD3 cells. Proc. Natl. Acad. Sci. U.S.A. **100**, 6428–6433 (2003). 12746499 10.1073/pnas.1130871100 PMC164463

[13] F. Roger, P.-Y. Martin, M. Rousselot, H. Favre, E. Féraille, Cell shrinkage triggers the activation of mitogen-activated protein kinases by hypertonicity in the rat kidney medullary thick ascending limb of the Henle’s loop. J. Biol. Chem. **274**, 34103–34110 (1999). 10567379 10.1074/jbc.274.48.34103

[14] J. Kim, R. P. Sharma, Calcium-mediated activation of c-Jun NH_2_-terminal kinase (JNK) and apoptosis in response to cadmium in murine macrophages. Toxicol. Sci. **81**, 518–527 (2004). 15254339 10.1093/toxsci/kfh221

[15] C. Kanellopoulou , Mg^2+^ regulation of kinase signaling and immune function. J. Exp. Med. **216**, 1828–1842 (2019). 31196981 10.1084/jem.20181970 PMC6683994

