# Supplementary material for: Proton-activated chloride channel PACC1 as acid sensor in epidermal desquamation
Source: Proc Natl Acad Sci U S A. 2026 Mar 31;123(14):e2601316123. doi: 10.1073/pnas.2601316123 (PMC13056114; doi:10.1073/pnas.2601316123)
Supplement: Supplementary file 1 — Appendix 01 (PDF) [file pnas.2601316123.sapp.pdf]

PNAS

Supporting Information for

Proton-activated chloride channel PACC1 as acid sensor in epidermal desquamation

Keyu Xia[a,1], Xiangjian Liu[a,1], Jiajing Wu[a,1], Junyan Hu[a], Xufeng Cheng[a], Tingyan Mi[b], Bomin Gao[a], Xiaoyi Mo[a], Xuteng Lu[a], Feng Cao[b], Chang Xie[a], Jian Cao[b], Nadine Pernodet[c], Guangwen He[b], Huanjun Zhou[b]*, Jing Yao[a]*

[a] State Key Laboratory of Virology and Biosafety, Hubei Provincial Research Center for Basic Biological Sciences, College of Life Sciences, TaiKang Center for Life and Medical Sciences, Hubei Key Laboratory of Cell Homeostasis, Frontier Science Center for Immunology and Metabolism, Wuhan University, Wuhan, Hubei 430072, China

[b] Estée Lauder Companies Innovation R&D (China) Co. Ltd., Shanghai, 200233, China.

[c] Global Research and Development, The Estée Lauder Companies, Melville, New York, USA

[1] These authors contributed equally to this work.

* Corresponding author: Jing Yao and Huanjun Zhou

Email: jyao@whu.edu.cn (J.Y.), huazhou@cn.estee.com (H.Z.)

This PDF file includes:

Supporting text

Supporting Information Text

Extended Materials and Methods

cDNA constructs, Cell culture and Gene expression

Full-length cDNAs encoding human PACC1 (NM_018252.3), JUN (NM_002228.4) and c-fos (NM_005252.4) were isolated and cloned from the human genome. All mutations were generated using the overlap-extension polymerase chain reaction (PCR) method. Primers were ordered from Sangon Biotech. The resulting mutations were then verified by DNA sequencing. Human Keratinocytes and HaCaT cell line were purchased from Biocell (Shan'xi, China), authenticated by STR locus and tested negative for mycoplasma contamination. HaCaT cells were maintained in Dulbecco's Modified Eagle's medium (DMEM, Thermo Fisher Scientific, USA) containing 4.5 mg/ml glucose, with 10% heat-inactivated fetal bovine serum (FBS) and 1% penicillin/streptomycin. Primary human keratinocytes were maintained in DermaLife Basal Medium (Lifeline Cell Technology, Frederick, MD, USA) supplemented with DermaLife K LifeFactors (Lifeline Cell Technology). All cells were incubated at 37°C in a humidified incubator gassed with 5% CO_2. Cells grown to ~80% confluence were transfected with the desired DNA constructs using Lipofectamine 2000 (Invitrogen, Carlsbad, CA) according to the manufacturer's instruction. Transfected cells were then reseeded on 12-mm round glass coverslips coated with poly-L-lysine. Subsequent experiments were performed 24-48 hours after transfection.

Electrophysiological recording

Conventional whole-cell patch-clamp recording method was used. For the recombinant expression system, green fluorescent EGFP was used as a surface marker for gene expression. Patch-clamp recordings were voltage-clamped using an Axopatch 200B amplifier (Molecular Devices, Sunnyvale, CA) and recorded through a BNC-2090/MIO acquisition system (National Instruments, Austin, TX). Data acquisition was controlled by QStudio, which was developed by Dr. Feng Qin at the State University of New York at Buffalo. Recording pipettes were pulled from borosilicate glass capillaries (World Precision Instruments), and fire-polished to a resistance between 2-4 MΩ when filled with an internal solution. Data were typically sampled at 5 kHz and low-pass filtered at 1 kHz. The compensation of pipette series resistance and capacitance were compensated (>80%) using the built-in circuitry of the amplifier to reduce voltage errors. For whole-cell recordings, the bath solution contained (in mM): 140 NaCl, 5 KCl, 3 EGTA, and 10 HEPES, pH 7.4 adjusted with NaOH. The internal pipette solution consisted (in mM): 145 CsCl, 5 EGTA and 10 HEPES, pH 7.4 (adjusted with CsOH). The acidic solutions contained (in mM): 140 NaCl, 5 KCl, 3 EGTA, and 15 Citric Acid, with pH adjusted to the desired values by NaOH. Unless otherwise stated, all chemicals were purchased from Sigma (Sigma, St Louis, MO). Water-insoluble reagents were dissolved in either 100% ethanol or DMSO to make stock solutions and were diluted in the recording solutions at appropriate concentrations before experiments. The final concentrations of ethanol or DMSO did not exceed 0.3%, which did not affect the currents. Exchange of external solution was performed using a gravity-driven local perfusion system. As determined by the conductance tests, the solution around a patch under study was completely controlled by the application of a flow rate of 100 μl/min or greater. All experiments were performed at room temperature (22-24°C).

Western blot

Briefly, cells were collected and lysed in Nonidet P-40 lysis buffer containing 150 mM NaCl, 1 mM EDTA, 1% Nonidet P-40, 1% protease inhibitor cocktail, and 1% phosphatase inhibitor cocktail, if needed, after washing with PBS. Then, the anti-Flag affinity gel or the appropriate antibodies were added to the lysates and incubated at 4°C for 4 hr or overnight with slow rotation. After washing three times with prelysis buffer containing 500 mM NaCl, the precipitates were resuspended in 5× SDS sample buffer, boiled, and subjected to SDS-polyacrylamide gel electrophoresis (SDS-PAGE). Immunoblot analysis was then performed using the appropriate antibodies.

Enzyme-linked immunosorbent assay

After stimulating the cells under different conditions and culturing them for 24 hours, the cell culture medium was collected. The samples were then centrifuged at 3,000 rpm for 10 minutes to remove particles and polymers. The levels of secreted KLK5/7 were quantified by ELISA kits (HYCEZMBIO, HYCYX201210/HYCYX201211). Absorbance (OD value) was measured at 450 nm using a Cytation 3 microplate reader (BioTek, USA), and sample concentrations were determined relative to the control group.

Establishment of PACC1 knockdown cell lines

Short hairpin RNA sequences targeting PACC1 were cloned into the lentiviral vector pLKO.1 to generate PACC1 knockdown cell lines. Lentiviral particles were produced by transient transfection of this recombinant pLKO.1 vector into HEK293T cells. HaCaT cells were then infected with the packaged lentivirus. Fourty-eight hours after infection, the culture medium was replaced with Dulbecco's Modified Eagle Medium (DMEM) supplemented with 2 μg/ml puromycin. This selection step was performed to enrich for cells that had successfully integrated the shRNA-containing lentiviral construct, as puromycin resistance is encoded by the pLKO.1 vector.

Establishment of PACC1 knockout cell lines

PACC1 knockout (KO) cell lines were created using a lentivirus-based CRISPR-Cas9 system. Two single guide RNAs (sgRNAs) were designed to target the putative transmembrane (TM) domain of PACC1. This domain was selected as the target to ensure disruption of PACC1's membrane-localized function. The sgRNAs were cloned into a custom lentiviral plasmid that harbors the Cas9 nuclease gene under the control of the EF1α promoter. The plasmid also contains dual sgRNA expression cassettes (U6-sg1-H1-sg2-ef1a-cas9-T2A-GFP). The GFP reporter gene, which is linked via a T2A sequence-, allows for visualization of the transfected cells. The sequences of the cloned sgRNAs were verified by Sanger sequencing to confirm no off-target mutations in the sgRNA coding regions.

Lentiviral particles containing the CRISPR-Cas9-sgRNA plasmid were packaged and used to infect HaCaT cells in the presence of 10 μg/ml Polybrene, a reagent that enhances lentiviral attachment and infection efficiency.

To isolate single-cell clones, GFP^+ cells were sorted into 96-well plates via flow cytometry. The sorted clones were expanded in culture for 3-4 weeks to generate sufficient cell numbers for

downstream validation. Genotyping of each clonal cell line was performed via a target-site-specific polymerase chain reaction. Primers were designed to amplify the PACC1 TM domain region targeted by the sgRNAs. The resulting PCR amplicons were subjected to Sanger sequencing to detect frameshift mutations, which are a hallmark of successful gene knockout because frameshifts disrupt protein translation. Only clones with confirmed frameshift mutations in PACC1 alleles were considered valid knockout (KO) cell lines, ensuring complete loss of PACC1 expression.

Quantification and statistical analysis

Densitometry was performed using ImageJ software (National Institutes of Health, NIH) to quantitatively analyze the bands on western blot images. Electrophysiological data were analyzed offline using Qstudio developed by Dr. Feng Qin at State University of New York at Buffalo, Clampfit (Molecular Devices, Sunnyvale, CA), IGOR (Wavemetrics, Lake Oswego, OR, USA), and OriginPro (OriginLab Corporation, MA, USA). For concentration-response analysis, the modified Hill equation was used: $Y = A1 + (A2 - A1)/[1 + 10 \text{^}(\log EC_{50} - X) * n_H]$, in which EC_{50} is the half maximal effective concentration, and n_H is the Hill coefficient. Data are presented as mean ± standard error (SE). Statistical significance was assessed using an unpaired Student's t-test for two-group comparisons, or one-way analysis of variance (ANOVA) for comparisons involving three or more groups. The *n* value represents the number of biological replicates. Statistical significance is denoted by a *p* value less than 0.05 (*$p < 0.05$, **$p < 0.01$, ***$p < 0.001$).
